# Supplementary material for: Direct Cell Death Induced by CD20 Monoclonal Antibodies on B Cell Lymphoma Cells Revealed by New Protocols of Analysis
Source: Cancers (Basel). 2023 Feb 9;15(4):1109. doi: 10.3390/cancers15041109 (PMC9954594; doi:10.3390/cancers15041109)
Supplement: Supplementary file 1 [file cancers-15-01109-s001.zip › cancers-2174282-supplementary.pptx]

## Slide 1
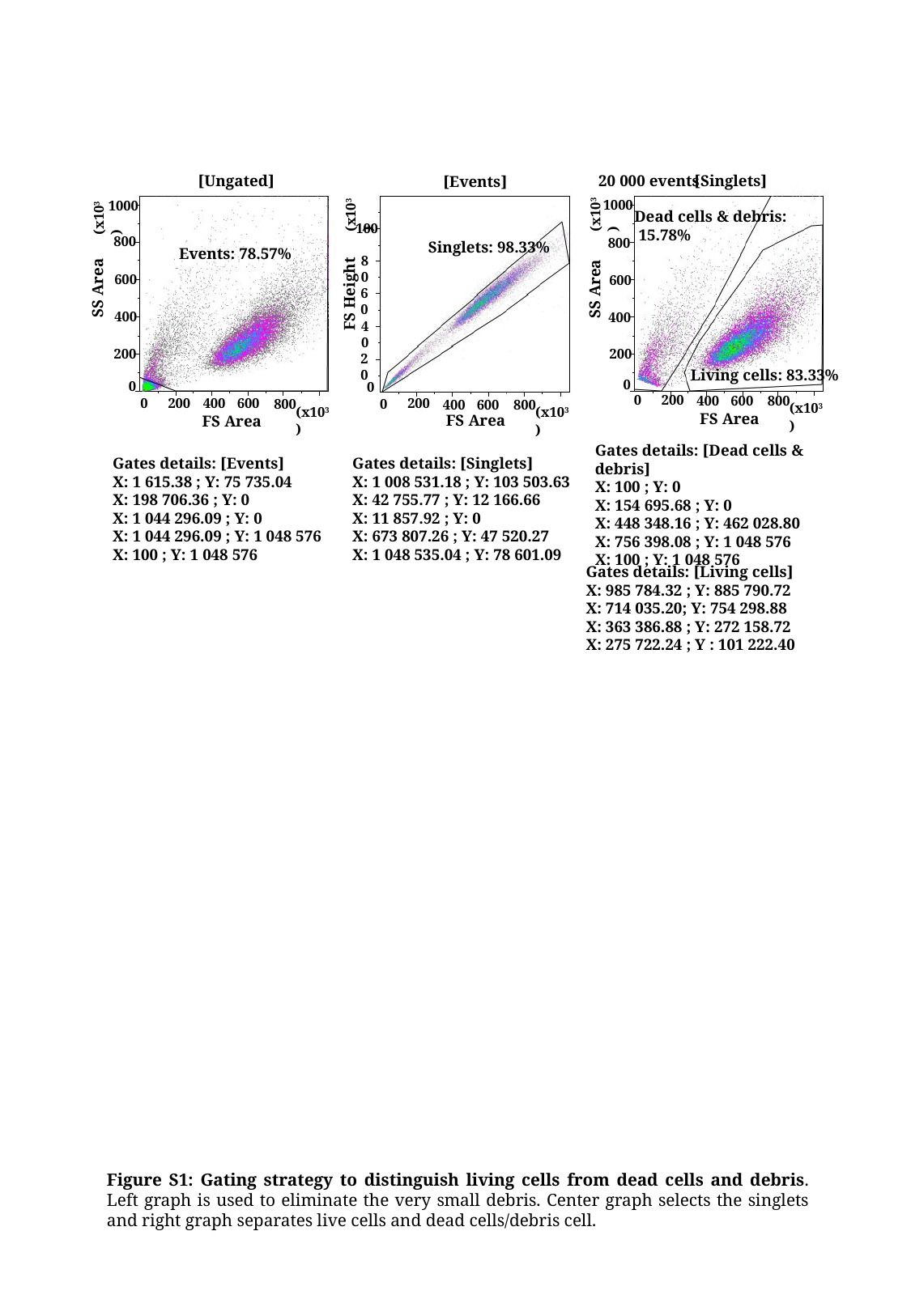

[Ungated]
1000
(x103)
800
600
400
200
0
Events: 78.57%
SS Area
200
0
400
600
800
(x103)
FS Area
20 000 events
[Singlets]
1000
(x103)
800
600
400
200
0
Dead cells & debris:
 15.78%
SS Area
Living cells: 83.33%
200
0
400
600
800
(x103)
FS Area
[Events]
(x103)
100
80
60
40
20
0
Singlets: 98.33%
FS Height
200
0
400
600
800
(x103)
FS Area
Gates details: [Dead cells & debris]
X: 100 ; Y: 0
X: 154 695.68 ; Y: 0
X: 448 348.16 ; Y: 462 028.80
X: 756 398.08 ; Y: 1 048 576
X: 100 ; Y: 1 048 576
Gates details: [Events]
X: 1 615.38 ; Y: 75 735.04
X: 198 706.36 ; Y: 0
X: 1 044 296.09 ; Y: 0
X: 1 044 296.09 ; Y: 1 048 576
X: 100 ; Y: 1 048 576
Gates details: [Singlets]
X: 1 008 531.18 ; Y: 103 503.63
X: 42 755.77 ; Y: 12 166.66
X: 11 857.92 ; Y: 0
X: 673 807.26 ; Y: 47 520.27
X: 1 048 535.04 ; Y: 78 601.09
Gates details: [Living cells]
X: 985 784.32 ; Y: 885 790.72
X: 714 035.20; Y: 754 298.88
X: 363 386.88 ; Y: 272 158.72
X: 275 722.24 ; Y : 101 222.40
Figure S1: Gating strategy to distinguish living cells from dead cells and debris. Left graph is used to eliminate the very small debris. Center graph selects the singlets and right graph separates live cells and dead cells/debris cell.

## Slide 2
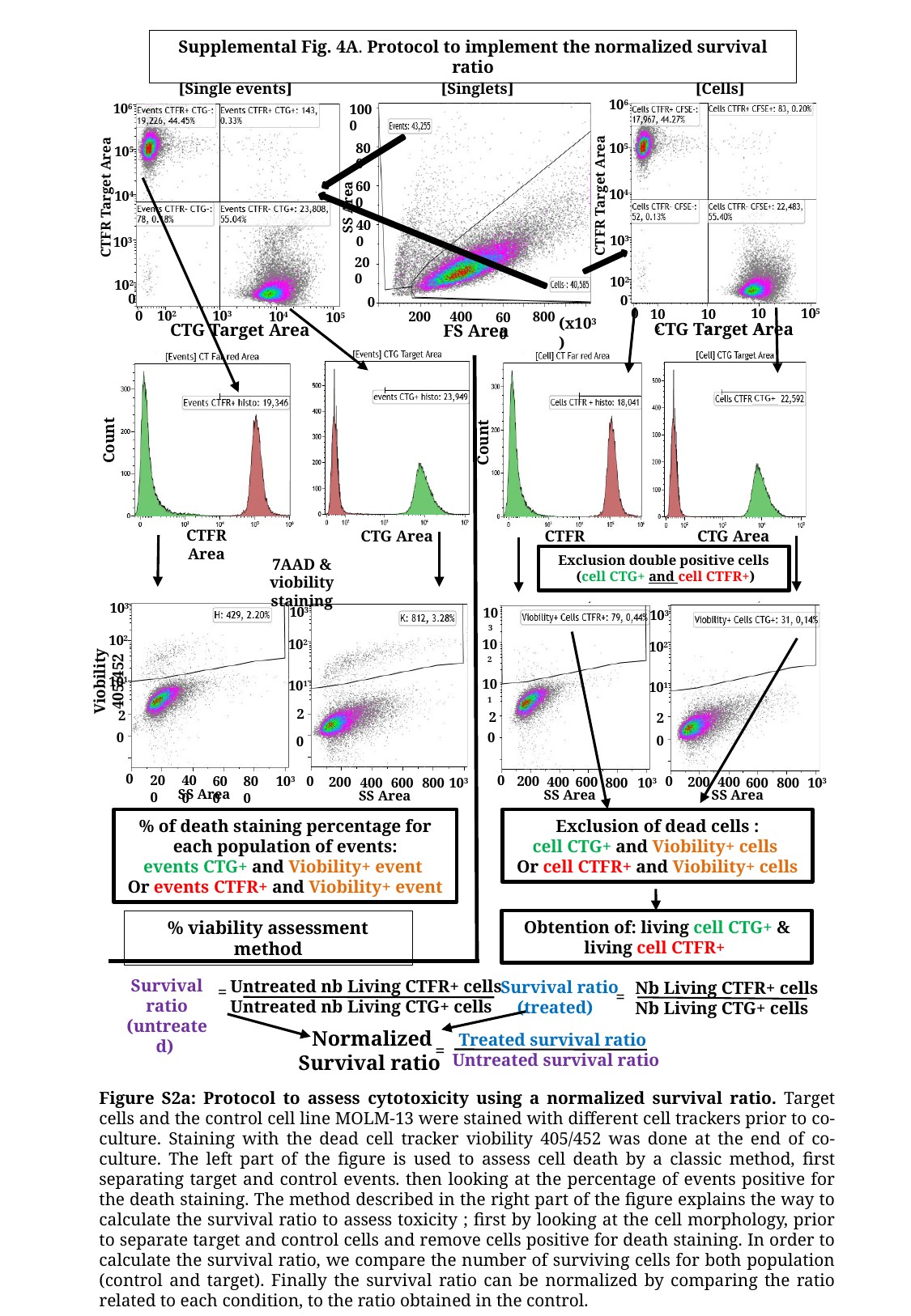

Supplemental Fig. 4A. Protocol to implement the normalized survival ratio
[Singlets]
1000
800
600
SS Area
400
0
200
200
400
800
600
(x103)
FS Area
[Single events]
[Cells]
106
105
104
CTFR Target Area
103
102
0
104
0
105
103
102
CTG Target Area
106
105
104
CTFR Target Area
103
102
0
0
102
103
104
105
CTG Target Area
Count
Count
CTFR Area
CTFR Area
CTG Area
CTG Area
Exclusion double positive cells
 (cell CTG+ and cell CTFR+)
7AAD & viobility staining
103
102
Viobility 405/452
101
0
2
0
200
400
103
600
800
SS Area
103
102
101
2
0
103
102
101
2
0
103
102
101
2
0
0
200
400
600
800
103
SS Area
0
200
400
103
600
800
SS Area
0
200
400
103
600
800
SS Area
% of death staining percentage for each population of events:
events CTG+ and Viobility+ event
Or events CTFR+ and Viobility+ event
Exclusion of dead cells :
cell CTG+ and Viobility+ cells
Or cell CTFR+ and Viobility+ cells
% viability assessment method
Obtention of: living cell CTG+ & living cell CTFR+
Survival ratio
(untreated)
Untreated nb Living CTFR+ cells
Untreated nb Living CTG+ cells
=
Survival ratio
(treated)
Nb Living CTFR+ cells
Nb Living CTG+ cells
=
Normalized
Survival ratio
Treated survival ratio
=
Untreated survival ratio
Figure S2a: Protocol to assess cytotoxicity using a normalized survival ratio. Target cells and the control cell line MOLM-13 were stained with different cell trackers prior to co-culture. Staining with the dead cell tracker viobility 405/452 was done at the end of co-culture. The left part of the figure is used to assess cell death by a classic method, first separating target and control events. then looking at the percentage of events positive for the death staining. The method described in the right part of the figure explains the way to calculate the survival ratio to assess toxicity ; first by looking at the cell morphology, prior to separate target and control cells and remove cells positive for death staining. In order to calculate the survival ratio, we compare the number of surviving cells for both population (control and target). Finally the survival ratio can be normalized by comparing the ratio related to each condition, to the ratio obtained in the control.

## Slide 3
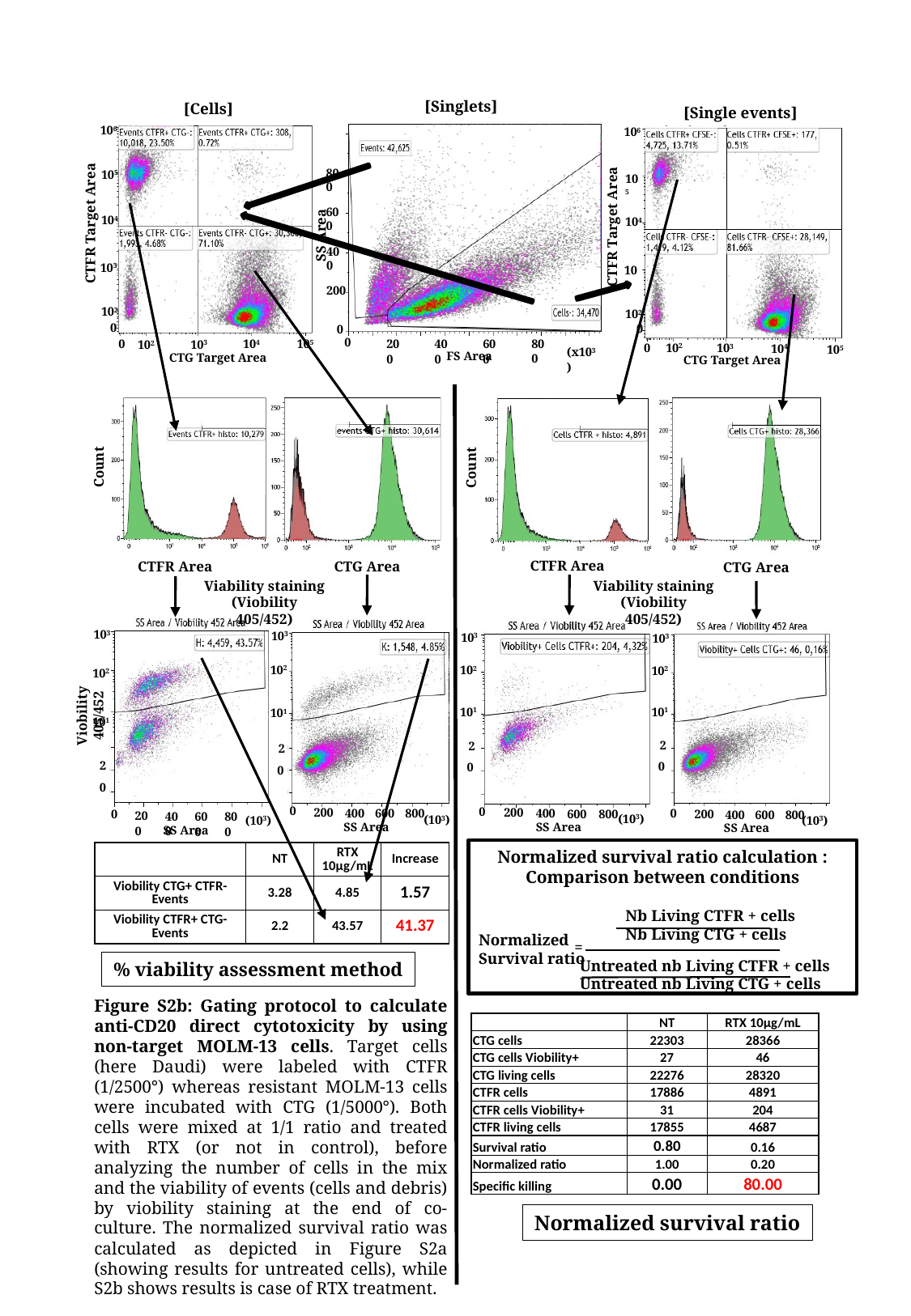

[Singlets]
800
600
SS Area
400
200
0
0
800
200
400
600
(x103)
FS Area
[Cells]
106
105
104
CTFR Target Area
103
102
0
0
104
105
103
102
CTG Target Area
[Single events]
106
105
104
CTFR Target Area
103
102
0
102
103
0
104
105
CTG Target Area
Count
Count
CTFR Area
CTFR Area
CTG Area
CTG Area
Viability staining (Viobility 405/452)
Viability staining (Viobility 405/452)
103
102
Viobility 405/452
101
2
0
0
200
400
600
800
SS Area
103
102
101
2
0
0
200
400
600
800
(103)
SS Area
103
102
101
2
0
0
200
400
600
800
SS Area
103
102
101
2
0
0
200
400
600
800
SS Area
(103)
(103)
(103)
Normalized survival ratio calculation : Comparison between conditions
Nb Living CTFR + cells
Nb Living CTG + cells
Normalized
Survival ratio
=
Untreated nb Living CTFR + cells
Untreated nb Living CTG + cells
| | NT | RTX 10µg/mL | Increase |
| --- | --- | --- | --- |
| Viobility CTG+ CTFR- Events | 3.28 | 4.85 | 1.57 |
| Viobility CTFR+ CTG- Events | 2.2 | 43.57 | 41.37 |
% viability assessment method
Figure S2b: Gating protocol to calculate anti-CD20 direct cytotoxicity by using non-target MOLM-13 cells. Target cells (here Daudi) were labeled with CTFR (1/2500°) whereas resistant MOLM-13 cells were incubated with CTG (1/5000°). Both cells were mixed at 1/1 ratio and treated with RTX (or not in control), before analyzing the number of cells in the mix and the viability of events (cells and debris) by viobility staining at the end of co-culture. The normalized survival ratio was calculated as depicted in Figure S2a (showing results for untreated cells), while S2b shows results is case of RTX treatment.
| | NT | RTX 10µg/mL |
| --- | --- | --- |
| CTG cells | 22303 | 28366 |
| CTG cells Viobility+ | 27 | 46 |
| CTG living cells | 22276 | 28320 |
| CTFR cells | 17886 | 4891 |
| CTFR cells Viobility+ | 31 | 204 |
| CTFR living cells | 17855 | 4687 |
| Survival ratio | 0.80 | 0.16 |
| Normalized ratio | 1.00 | 0.20 |
| Specific killing | 0.00 | 80.00 |
Normalized survival ratio

## Slide 4
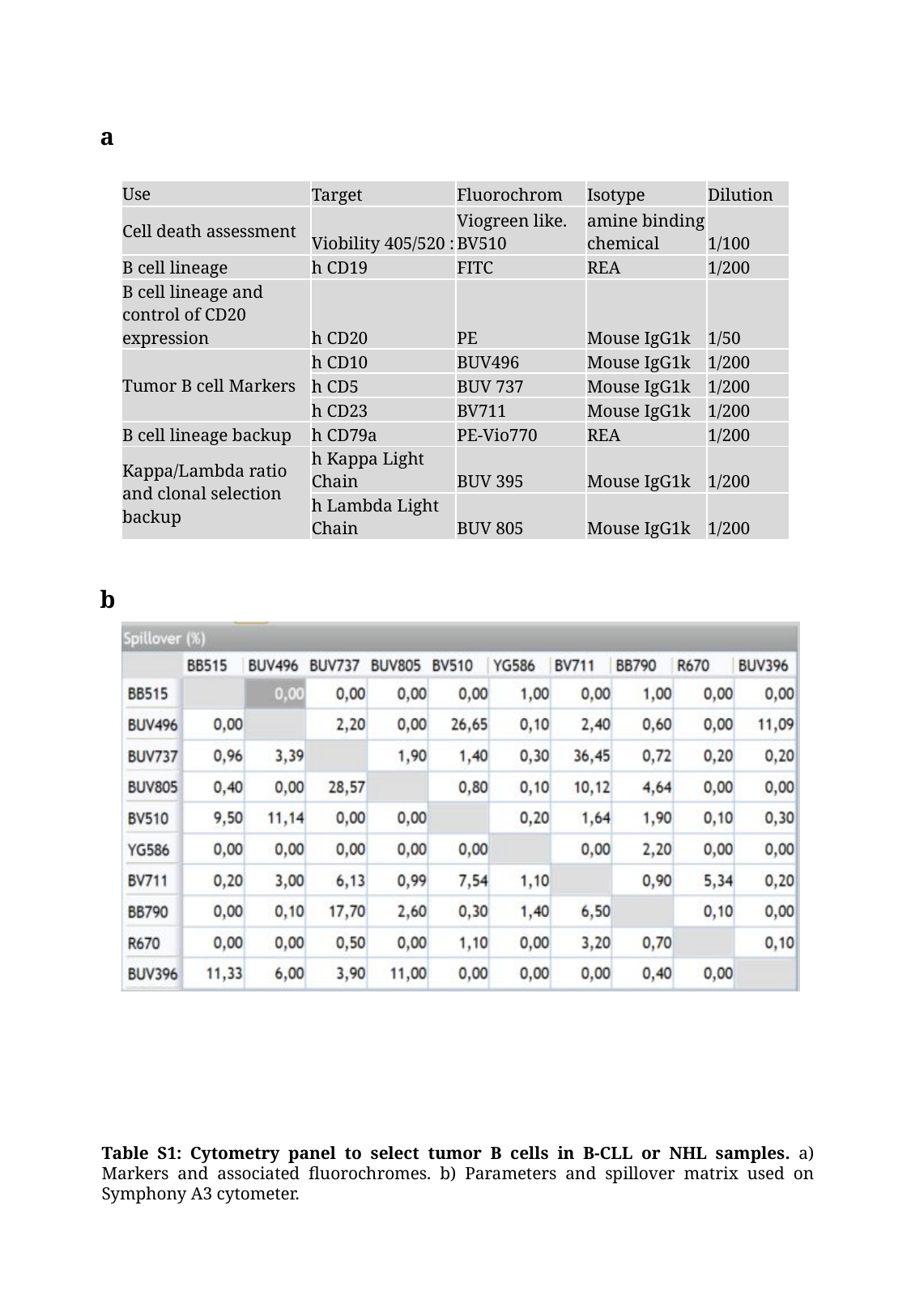

a
| Use | Target | Fluorochrom | Isotype | Dilution |
| --- | --- | --- | --- | --- |
| Cell death assessment | Viobility 405/520 : | Viogreen like. BV510 | amine binding chemical | 1/100 |
| B cell lineage | h CD19 | FITC | REA | 1/200 |
| B cell lineage and control of CD20 expression | h CD20 | PE | Mouse IgG1k | 1/50 |
| Tumor B cell Markers | h CD10 | BUV496 | Mouse IgG1k | 1/200 |
| | h CD5 | BUV 737 | Mouse IgG1k | 1/200 |
| | h CD23 | BV711 | Mouse IgG1k | 1/200 |
| B cell lineage backup | h CD79a | PE-Vio770 | REA | 1/200 |
| Kappa/Lambda ratio and clonal selection backup | h Kappa Light Chain | BUV 395 | Mouse IgG1k | 1/200 |
| | h Lambda Light Chain | BUV 805 | Mouse IgG1k | 1/200 |
b
Table S1: Cytometry panel to select tumor B cells in B-CLL or NHL samples. a) Markers and associated fluorochromes. b) Parameters and spillover matrix used on Symphony A3 cytometer.

## Slide 5
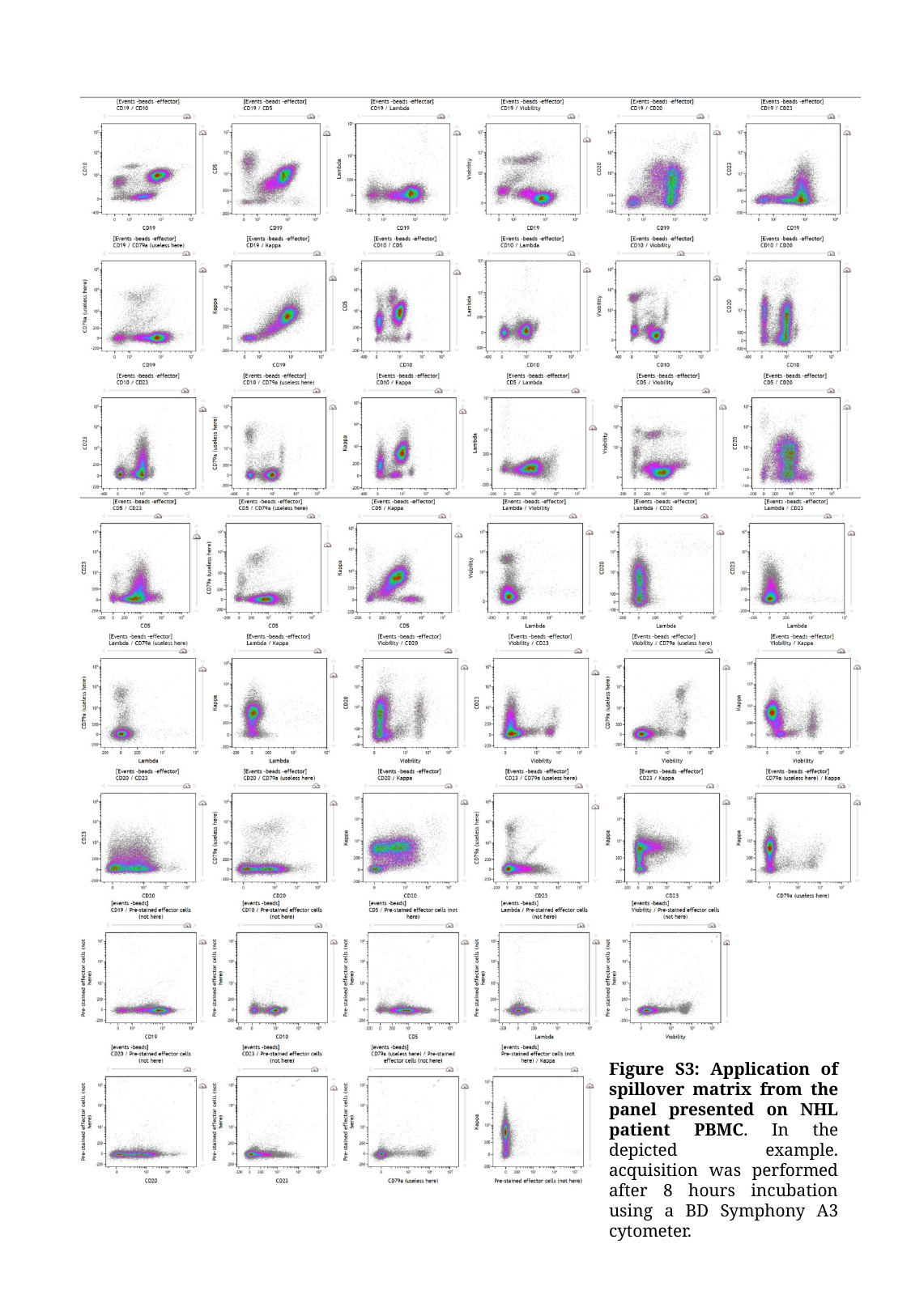

Figure S3: Application of spillover matrix from the panel presented on NHL patient PBMC. In the depicted example. acquisition was performed after 8 hours incubation using a BD Symphony A3 cytometer.

## Slide 6
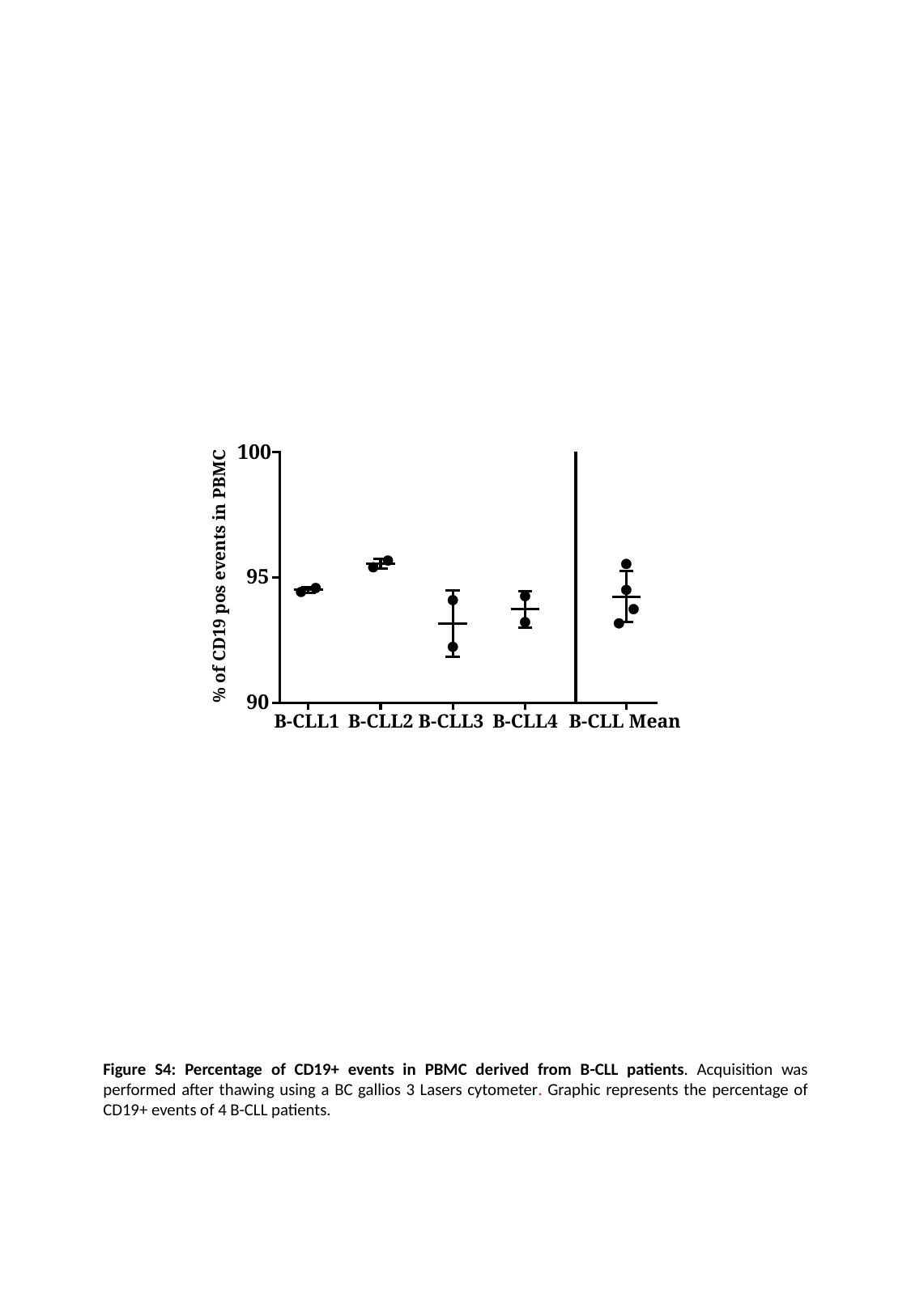

100
95
% of CD19 pos events in PBMC
90
B-CLL1
B-CLL2
B-CLL3
B-CLL4
B-CLL Mean
Figure S4: Percentage of CD19+ events in PBMC derived from B-CLL patients. Acquisition was performed after thawing using a BC gallios 3 Lasers cytometer. Graphic represents the percentage of CD19+ events of 4 B-CLL patients.

## Slide 7
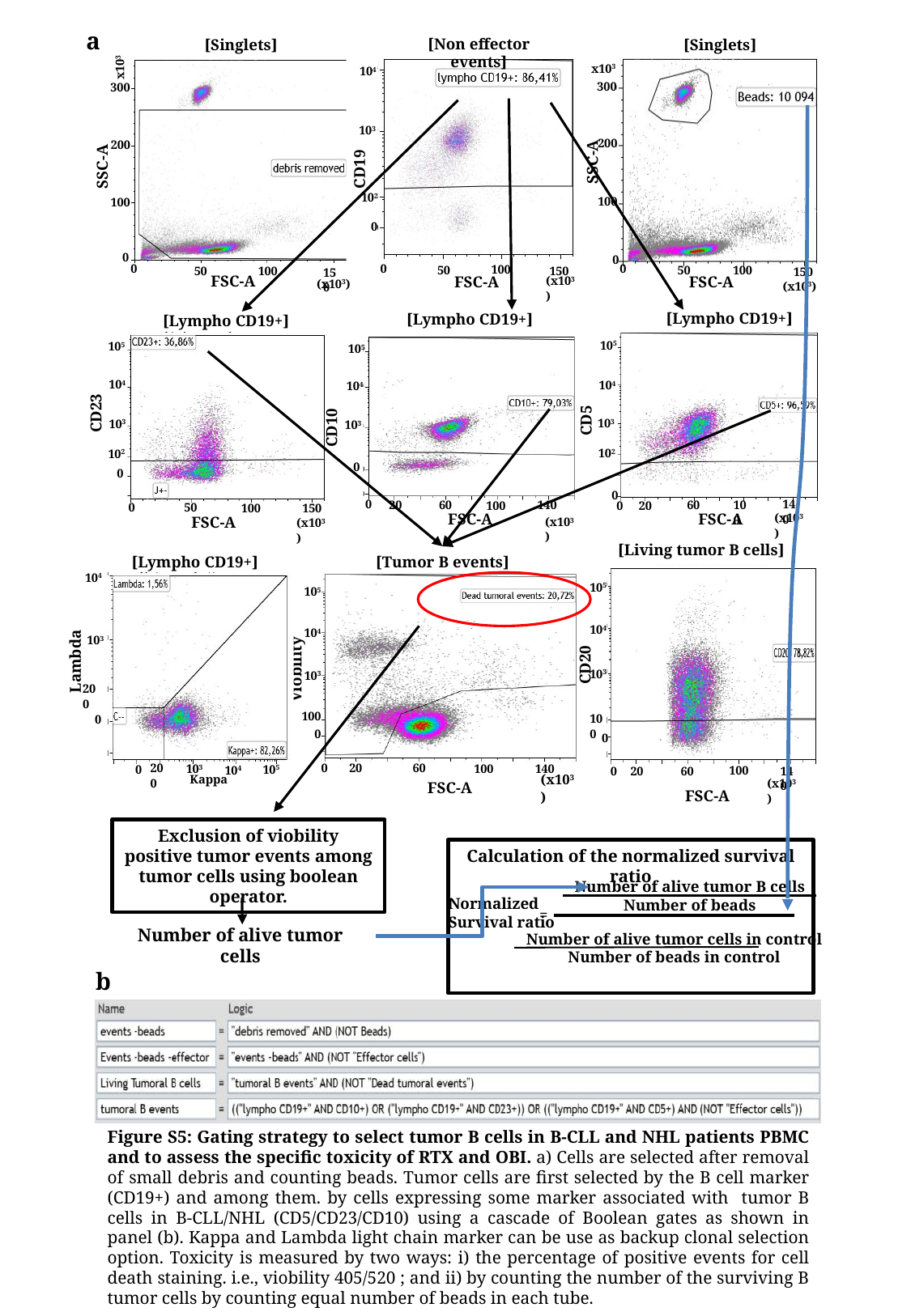

a
[Non effector events]
[Singlets]
[Singlets]
x103
300
200
SSC-A
100
0
0
100
50
150
FSC-A
(x103)
x103
300
200
100
0
0
50
100
150
104
103
102
0
0
100
50
150
(x103)
SSC-A
CD19
FSC-A
[Lympho CD19+]
105
104
103
102
0
0
50
150
100
(x103)
CD23
FSC-A
FSC-A
(x103)
[Lympho CD19+]
105
104
103
102
0
140
100
60
20
0
(x103)
CD5
FSC-A
[Lympho CD19+]
105
104
103
0
0
140
60
20
100
(x103)
CD10
FSC-A
[Living tumor B cells]
105
104
103
100
0
100
0
20
60
140
(x103)
CD20
FSC-A
[Tumor B events]
105
104
103
0
0
20
60
100
140
Viobility
(x103)
FSC-A
100
[Lympho CD19+]
104
103
200
0
200
103
105
0
104
Kappa
Lambda
Exclusion of viobility positive tumor events among tumor cells using boolean operator.
Calculation of the normalized survival ratio
Number of alive tumor B cells
Number of beads
Normalized
Survival ratio
=
Number of alive tumor cells in control
Number of beads in control
Number of alive tumor cells
b
Figure S5: Gating strategy to select tumor B cells in B-CLL and NHL patients PBMC and to assess the specific toxicity of RTX and OBI. a) Cells are selected after removal of small debris and counting beads. Tumor cells are first selected by the B cell marker (CD19+) and among them. by cells expressing some marker associated with tumor B cells in B-CLL/NHL (CD5/CD23/CD10) using a cascade of Boolean gates as shown in panel (b). Kappa and Lambda light chain marker can be use as backup clonal selection option. Toxicity is measured by two ways: i) the percentage of positive events for cell death staining. i.e., viobility 405/520 ; and ii) by counting the number of the surviving B tumor cells by counting equal number of beads in each tube.

## Slide 8
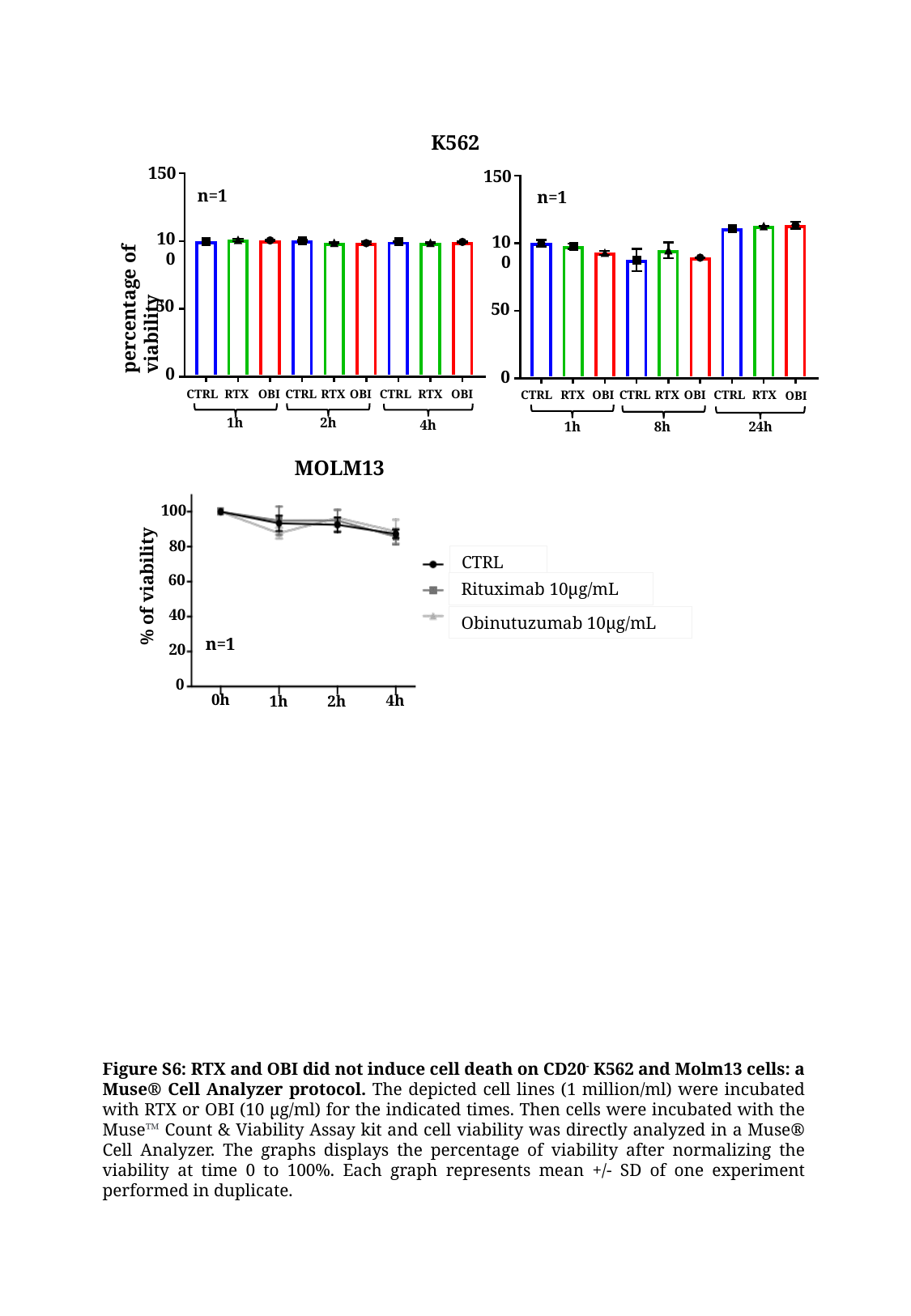

K562
150
100
50
0
n=1
percentage of viability
RTX
CTRL
OBI
OBI
RTX
CTRL
CTRL
OBI
RTX
1h
2h
4h
n=1
RTX
CTRL
OBI
RTX
CTRL
OBI
OBI
RTX
CTRL
8h
1h
24h
150
100
50
0
MOLM13
100
80
CTRL
% of viability
60
40
n=1
20
0
0h
4h
2h
1h
Rituximab 10µg/mL
Obinutuzumab 10µg/mL
Figure S6: RTX and OBI did not induce cell death on CD20- K562 and Molm13 cells: a Muse® Cell Analyzer protocol. The depicted cell lines (1 million/ml) were incubated with RTX or OBI (10 µg/ml) for the indicated times. Then cells were incubated with the Muse™ Count & Viability Assay kit and cell viability was directly analyzed in a Muse® Cell Analyzer. The graphs displays the percentage of viability after normalizing the viability at time 0 to 100%. Each graph represents mean +/- SD of one experiment performed in duplicate.

## Slide 9
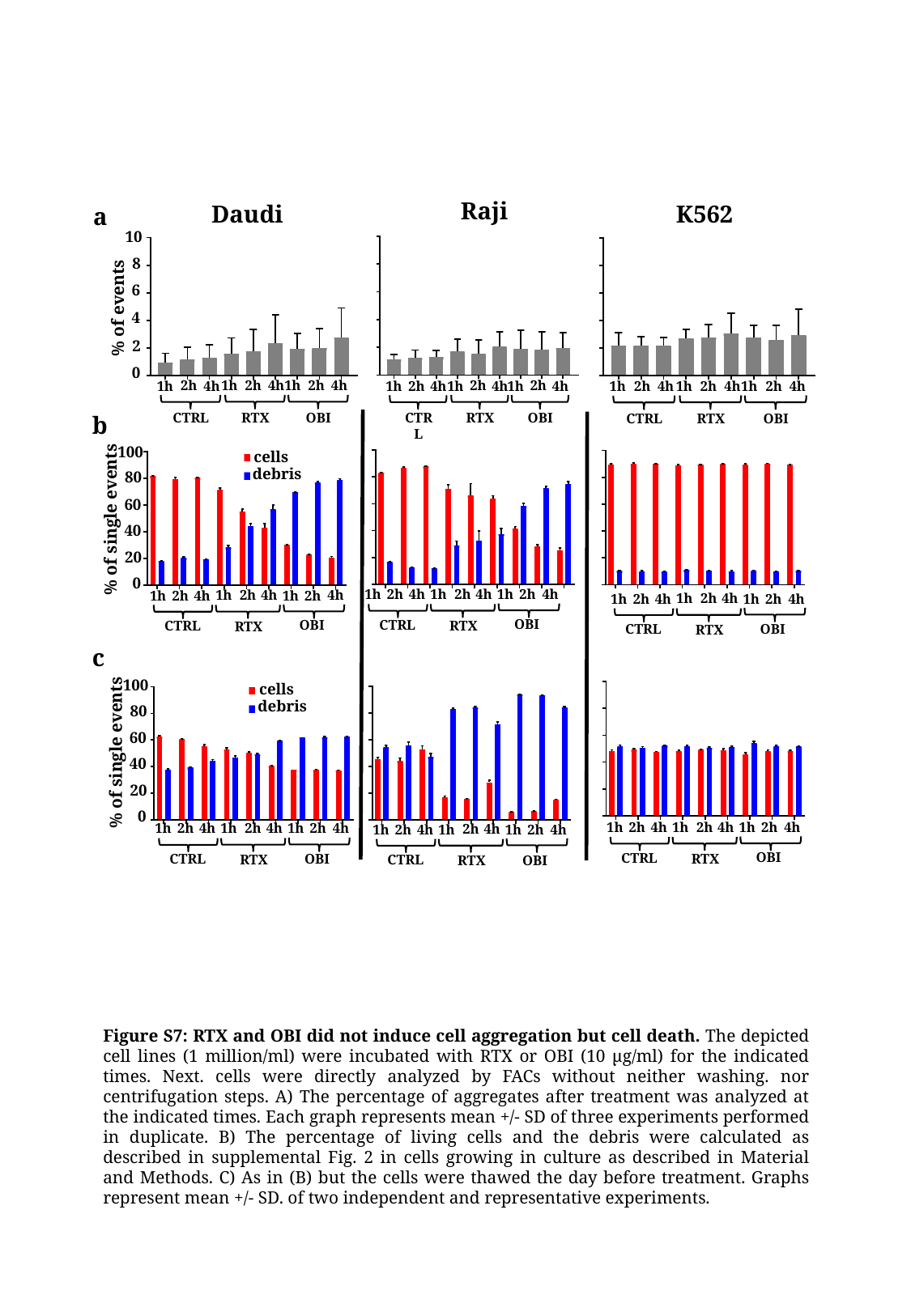

Raji
Daudi
K562
a
2h
1h
4h
2h
1h
4h
2h
1h
4h
RTX
OBI
CTRL
10
8
6
4
2
0
% of events
2h
1h
4h
2h
1h
4h
2h
1h
4h
RTX
OBI
CTRL
2h
1h
4h
2h
1h
4h
2h
1h
4h
RTX
OBI
CTRL
b
4h
2h
1h
4h
2h
1h
2h
4h
1h
OBI
CTRL
RTX
4h
2h
1h
4h
2h
1h
2h
4h
1h
OBI
CTRL
RTX
100
80
60
40
20
0
cells
debris
% of single events
4h
2h
1h
4h
2h
1h
2h
4h
1h
OBI
CTRL
RTX
c
4h
2h
1h
4h
2h
1h
2h
4h
1h
OBI
CTRL
RTX
4h
2h
1h
4h
2h
1h
2h
4h
1h
CTRL
RTX
OBI
100
80
60
40
20
0
cells
debris
% of single events
4h
2h
1h
4h
2h
1h
2h
4h
1h
OBI
CTRL
RTX
Figure S7: RTX and OBI did not induce cell aggregation but cell death. The depicted cell lines (1 million/ml) were incubated with RTX or OBI (10 µg/ml) for the indicated times. Next. cells were directly analyzed by FACs without neither washing. nor centrifugation steps. A) The percentage of aggregates after treatment was analyzed at the indicated times. Each graph represents mean +/- SD of three experiments performed in duplicate. B) The percentage of living cells and the debris were calculated as described in supplemental Fig. 2 in cells growing in culture as described in Material and Methods. C) As in (B) but the cells were thawed the day before treatment. Graphs represent mean +/- SD. of two independent and representative experiments.

## Slide 10
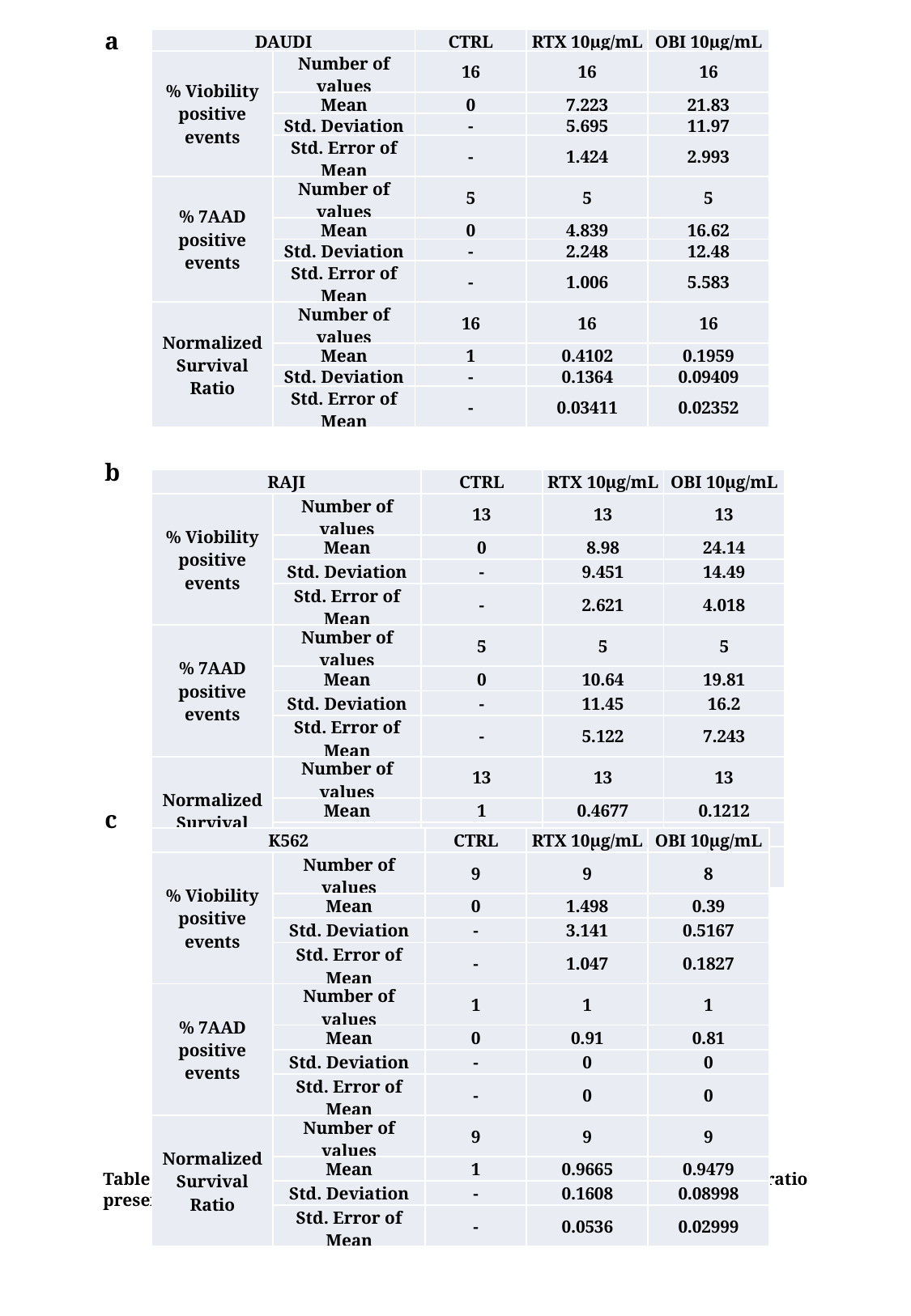

a
| DAUDI | | CTRL | RTX 10µg/mL | OBI 10µg/mL |
| --- | --- | --- | --- | --- |
| % Viobility positive events | Number of values | 16 | 16 | 16 |
| | Mean | 0 | 7.223 | 21.83 |
| | Std. Deviation | - | 5.695 | 11.97 |
| | Std. Error of Mean | - | 1.424 | 2.993 |
| % 7AAD positive events | Number of values | 5 | 5 | 5 |
| | Mean | 0 | 4.839 | 16.62 |
| | Std. Deviation | - | 2.248 | 12.48 |
| | Std. Error of Mean | - | 1.006 | 5.583 |
| Normalized Survival Ratio | Number of values | 16 | 16 | 16 |
| | Mean | 1 | 0.4102 | 0.1959 |
| | Std. Deviation | - | 0.1364 | 0.09409 |
| | Std. Error of Mean | - | 0.03411 | 0.02352 |
b
| RAJI | | CTRL | RTX 10µg/mL | OBI 10µg/mL |
| --- | --- | --- | --- | --- |
| % Viobility positive events | Number of values | 13 | 13 | 13 |
| | Mean | 0 | 8.98 | 24.14 |
| | Std. Deviation | - | 9.451 | 14.49 |
| | Std. Error of Mean | - | 2.621 | 4.018 |
| % 7AAD positive events | Number of values | 5 | 5 | 5 |
| | Mean | 0 | 10.64 | 19.81 |
| | Std. Deviation | - | 11.45 | 16.2 |
| | Std. Error of Mean | - | 5.122 | 7.243 |
| Normalized Survival Ratio | Number of values | 13 | 13 | 13 |
| | Mean | 1 | 0.4677 | 0.1212 |
| | Std. Deviation | - | 0.2494 | 0.07551 |
| | Std. Error of Mean | - | 0.06917 | 0.02094 |
c
| K562 | | CTRL | RTX 10µg/mL | OBI 10µg/mL |
| --- | --- | --- | --- | --- |
| % Viobility positive events | Number of values | 9 | 9 | 8 |
| | Mean | 0 | 1.498 | 0.39 |
| | Std. Deviation | - | 3.141 | 0.5167 |
| | Std. Error of Mean | - | 1.047 | 0.1827 |
| % 7AAD positive events | Number of values | 1 | 1 | 1 |
| | Mean | 0 | 0.91 | 0.81 |
| | Std. Deviation | - | 0 | 0 |
| | Std. Error of Mean | - | 0 | 0 |
| Normalized Survival Ratio | Number of values | 9 | 9 | 9 |
| | Mean | 1 | 0.9665 | 0.9479 |
| | Std. Deviation | - | 0.1608 | 0.08998 |
| | Std. Error of Mean | - | 0.0536 | 0.02999 |
Table S2: Results and statistics of mortality and normalized survival ratio presented in figure 4. Statistics were performed using two tailed paired t-test.

## Slide 11
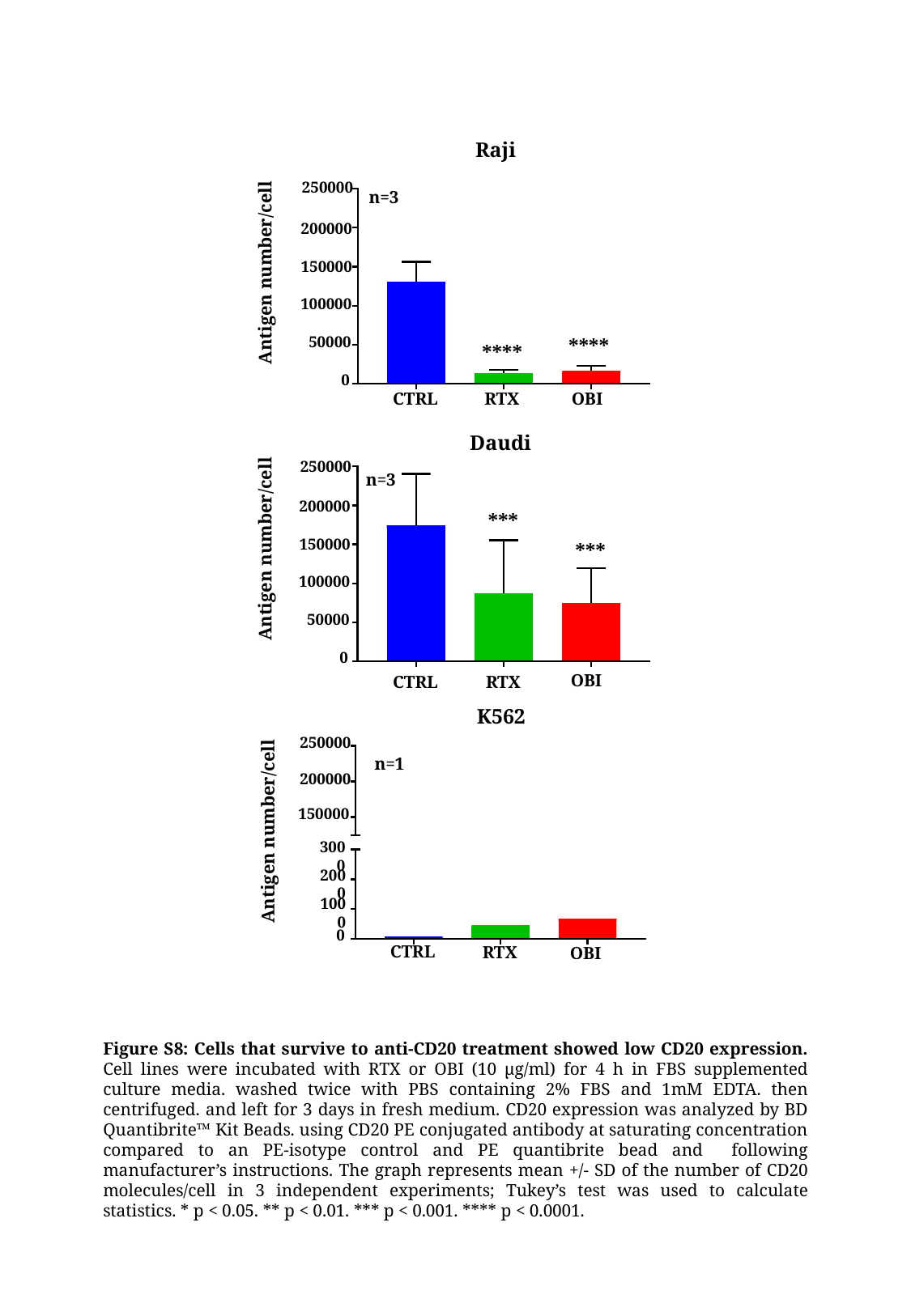

Raji
250000
200000
150000
100000
50000
0
n=3
Antigen number/cell
****
****
RTX
CTRL
OBI
Daudi
250000
200000
150000
100000
50000
0
n=3
***
Antigen number/cell
***
OBI
RTX
CTRL
K562
250000
200000
150000
n=1
Antigen number/cell
3000
2000
1000
0
CTRL
RTX
OBI
Figure S8: Cells that survive to anti-CD20 treatment showed low CD20 expression. Cell lines were incubated with RTX or OBI (10 µg/ml) for 4 h in FBS supplemented culture media. washed twice with PBS containing 2% FBS and 1mM EDTA. then centrifuged. and left for 3 days in fresh medium. CD20 expression was analyzed by BD Quantibrite™ Kit Beads. using CD20 PE conjugated antibody at saturating concentration compared to an PE-isotype control and PE quantibrite bead and following manufacturer’s instructions. The graph represents mean +/- SD of the number of CD20 molecules/cell in 3 independent experiments; Tukey’s test was used to calculate statistics. * p < 0.05. ** p < 0.01. *** p < 0.001. **** p < 0.0001.

## Slide 12
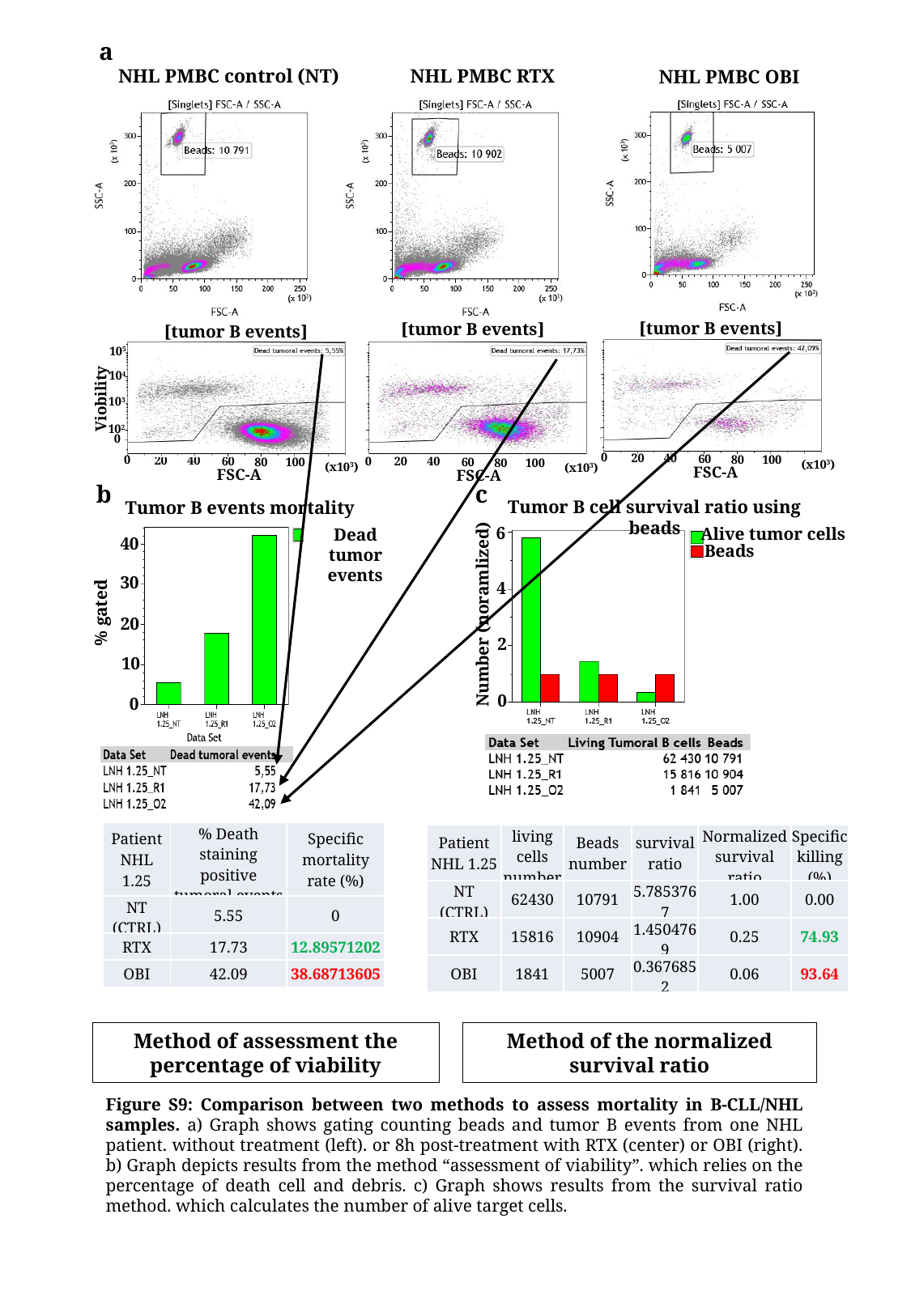

a
NHL PMBC control (NT)
NHL PMBC RTX
NHL PMBC OBI
[tumor B events]
[tumor B events]
[tumor B events]
105
104
103
102
0
0
Viobility
20
40
60
80
100
(x103)
FSC-A
0
20
40
60
80
100
(x103)
FSC-A
20
40
60
80
100
(x103)
FSC-A
0
b
c
Tumor B cell survival ratio using beads
Tumor B events mortality
6
Alive tumor cells
Dead tumor events
40
Beads
30
4
% gated
Number (noramlized)
20
2
10
0
0
| Patient NHL 1.25 | % Death staining positive tumoral events | Specific mortality rate (%) |
| --- | --- | --- |
| NT (CTRL) | 5.55 | 0 |
| RTX | 17.73 | 12.89571202 |
| OBI | 42.09 | 38.68713605 |
| Patient NHL 1.25 | living cells number | Beads number | survival ratio | Normalized survival ratio | Specific killing (%) |
| --- | --- | --- | --- | --- | --- |
| NT (CTRL) | 62430 | 10791 | 5.7853767 | 1.00 | 0.00 |
| RTX | 15816 | 10904 | 1.4504769 | 0.25 | 74.93 |
| OBI | 1841 | 5007 | 0.3676852 | 0.06 | 93.64 |
Method of assessment the percentage of viability
Method of the normalized survival ratio
Figure S9: Comparison between two methods to assess mortality in B-CLL/NHL samples. a) Graph shows gating counting beads and tumor B events from one NHL patient. without treatment (left). or 8h post-treatment with RTX (center) or OBI (right). b) Graph depicts results from the method “assessment of viability”. which relies on the percentage of death cell and debris. c) Graph shows results from the survival ratio method. which calculates the number of alive target cells.

## Slide 13
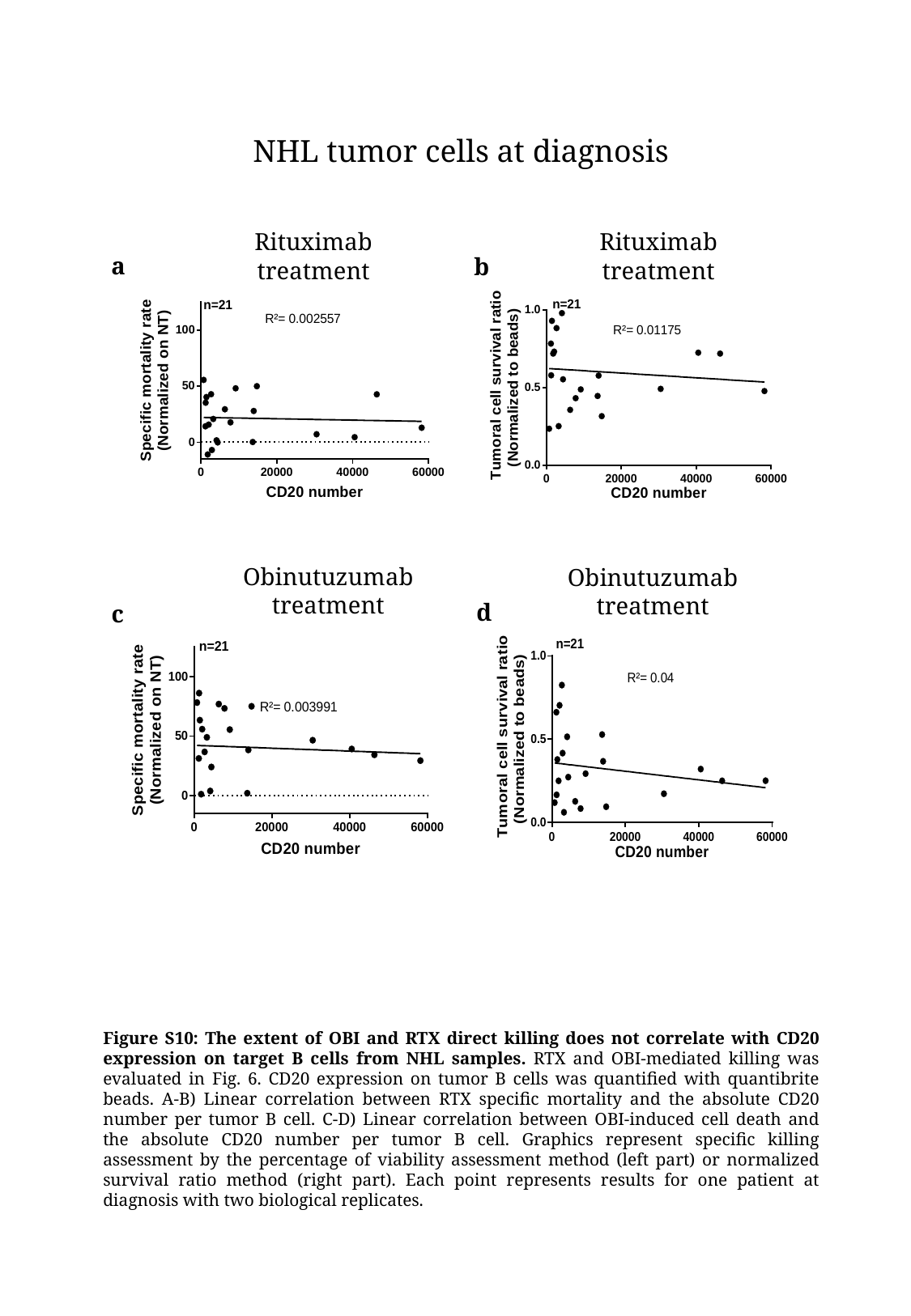

NHL tumor cells at diagnosis
Rituximab treatment
Rituximab treatment
a
b
Obinutuzumab treatment
Obinutuzumab treatment
d
c
Figure S10: The extent of OBI and RTX direct killing does not correlate with CD20 expression on target B cells from NHL samples. RTX and OBI-mediated killing was evaluated in Fig. 6. CD20 expression on tumor B cells was quantified with quantibrite beads. A-B) Linear correlation between RTX specific mortality and the absolute CD20 number per tumor B cell. C-D) Linear correlation between OBI-induced cell death and the absolute CD20 number per tumor B cell. Graphics represent specific killing assessment by the percentage of viability assessment method (left part) or normalized survival ratio method (right part). Each point represents results for one patient at diagnosis with two biological replicates.

## Slide 14
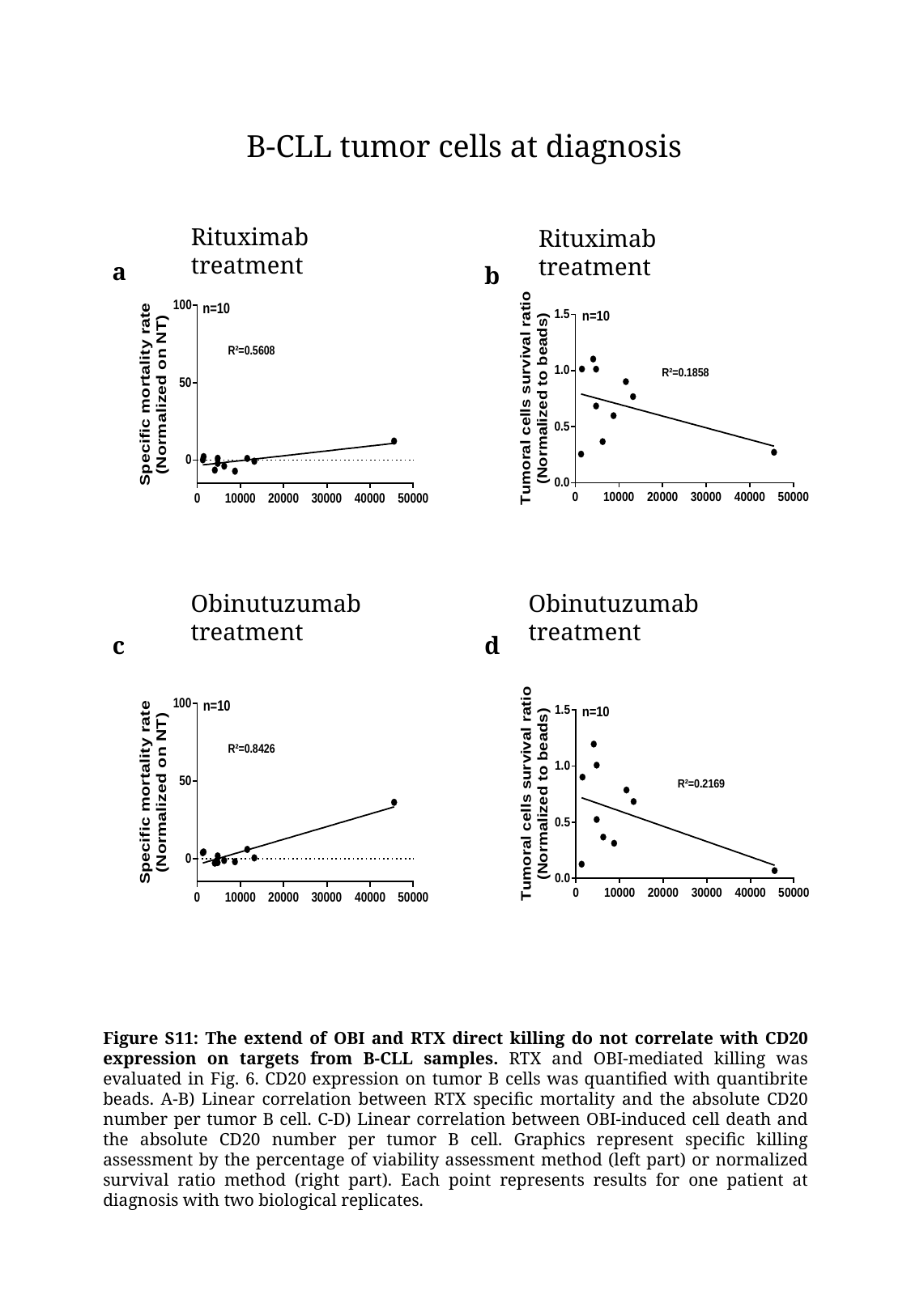

B-CLL tumor cells at diagnosis
Rituximab treatment
Rituximab treatment
a
b
Obinutuzumab treatment
Obinutuzumab treatment
c
d
Figure S11: The extend of OBI and RTX direct killing do not correlate with CD20 expression on targets from B-CLL samples. RTX and OBI-mediated killing was evaluated in Fig. 6. CD20 expression on tumor B cells was quantified with quantibrite beads. A-B) Linear correlation between RTX specific mortality and the absolute CD20 number per tumor B cell. C-D) Linear correlation between OBI-induced cell death and the absolute CD20 number per tumor B cell. Graphics represent specific killing assessment by the percentage of viability assessment method (left part) or normalized survival ratio method (right part). Each point represents results for one patient at diagnosis with two biological replicates.

## Slide 15
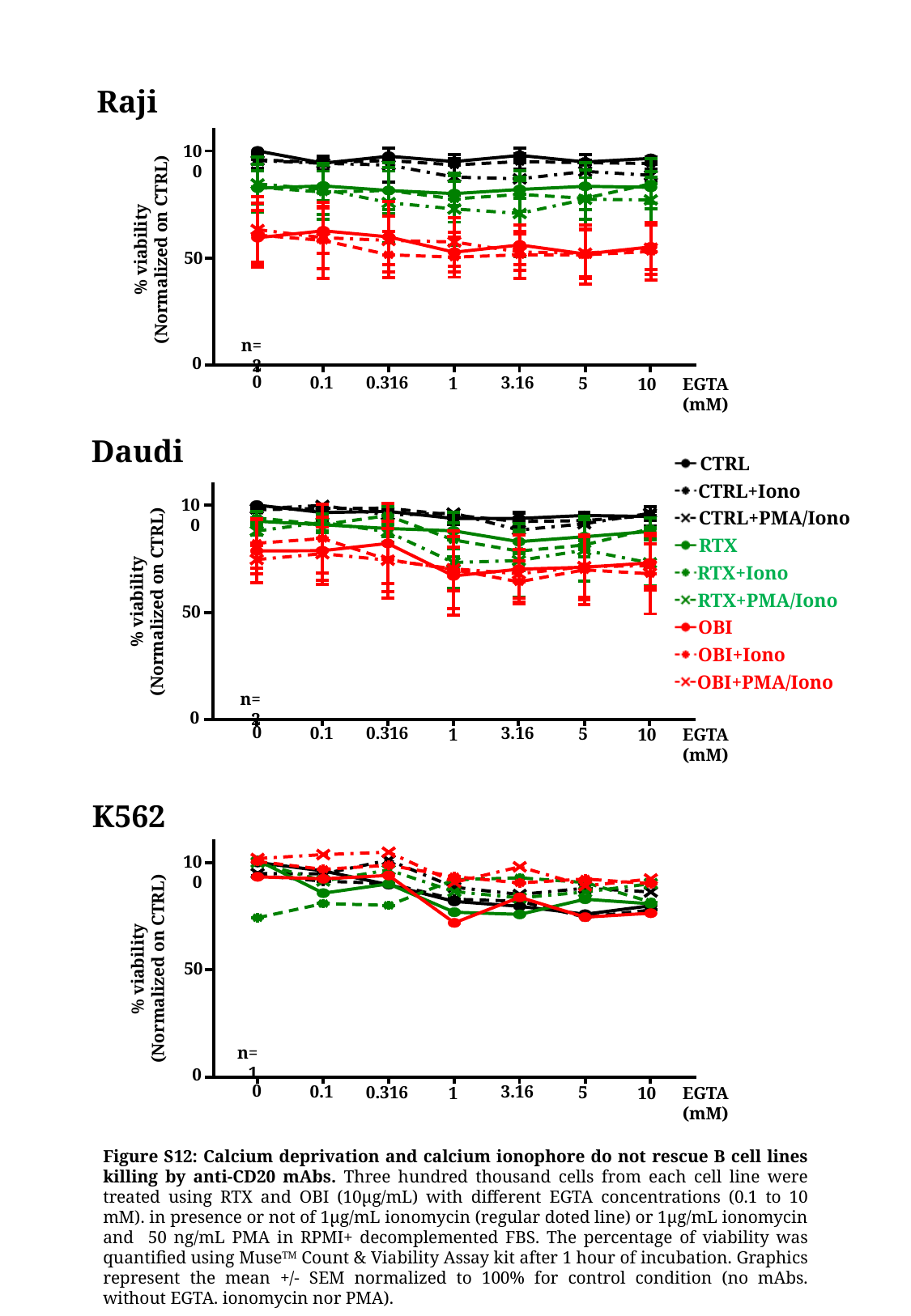

Raji
100
% viability
(Normalized on CTRL)
50
n=2
0
0
3.16
0.1
0.316
5
1
EGTA (mM)
10
Daudi
100
% viability
(Normalized on CTRL)
50
n=2
0
0
3.16
0.1
0.316
5
1
EGTA (mM)
10
CTRL
CTRL+Iono
CTRL+PMA/Iono
RTX
RTX+Iono
RTX+PMA/Iono
OBI
OBI+Iono
OBI+PMA/Iono
K562
100
% viability
(Normalized on CTRL)
50
n=1
0
0
3.16
0.1
0.316
5
1
10
EGTA (mM)
Figure S12: Calcium deprivation and calcium ionophore do not rescue B cell lines killing by anti-CD20 mAbs. Three hundred thousand cells from each cell line were treated using RTX and OBI (10µg/mL) with different EGTA concentrations (0.1 to 10 mM). in presence or not of 1µg/mL ionomycin (regular doted line) or 1µg/mL ionomycin and 50 ng/mL PMA in RPMI+ decomplemented FBS. The percentage of viability was quantified using MuseTM Count & Viability Assay kit after 1 hour of incubation. Graphics represent the mean +/- SEM normalized to 100% for control condition (no mAbs. without EGTA. ionomycin nor PMA).
